# A Theoretical and Empirical Linkage between Road Accidents and Binge Eating Behaviors in Adolescence

**DOI:** 10.3390/ijerph15020355

**Published:** 2018-02-17

**Authors:** Silvia Cimino, Alessandra Simonelli, Micol Parolin, Giulia Ballarotto, Paola Carbone, Luca Cerniglia

**Affiliations:** 1 Department of Dynamic and Clinical Psychology, University of Rome “Sapienza”, Rome 00100, Italy; silvia.cimino@uniroma1.it (S.C.); giulia.ballarotto@uniroma1.it (G.B.); paola.carbone@uniroma1.it (P.C.); 2 Department of Developmental and Social Psychology, University of Padua, Padua 35100, Italy; alessandra.simonelli@unipd.it (A.S.); micol.parolin@unipd.it (M.P.); 3 Department of Psychology, International Telematic University, Rome 00100, Italy

**Keywords:** adolescence, motorbike collisions, binge eating symptoms, alexithymia, impulsivity

## Abstract

This study aimed at identifying specific clusters of maladaptive emotional–behavioral symptoms in adolescent victims of motorbike collisions considering their scores on alexithymia and impulsivity in addition to examining the prevalence of clinical binge eating behaviors (respectively through the Youth Self-Report (YSR), Toronto Alexithymia Scale-20 (TAS-20), Barratt Impulsiveness Scale-11 (BIS-11), and Binge Eating Scale (BES)). Emotional–behavioral profiles, difficulties in identifying and describing feelings, impulsivity, and binge eating behaviors have been assessed in 159 adolescents addressing emergency departments following motorbike collisions. Our results showed a cluster of adolescents with clinical binge eating behaviors, high rates of motorbike accidents, and high levels of internalizing and externalizing problems, alexithymia, and impulsivity (23.3% of the sample); a second cluster of adolescents with clinical binge eating behaviors, a moderate number of collisions, and moderate levels of emotional and behavioral problems on the above four dimensions (25.8% of the sample); and a third cluster of youth without clinical binge eating behaviors, with a moderate number of accidents, and with low scores on the four dimensions (50.9% of the sample). Adolescents of Cluster 1 showed a higher likelihood to be involved in motorbike collisions than the youth in Clusters 2 and 3 (*p* < 0.0001). We suggest that adolescents’ motor collisions could be associated with their difficulties in emotion regulation and with their impaired psychological profiles, which could also underpin their disordered eating. The identification of specific clusters of psychopathological symptoms among this population could be useful for the construction of prevention and intervention programs aimed at reducing motor collision recidivism and alleviating co-occurring psychopathologies.

## 1. Introduction

It has been widely demonstrated that the prevalence of mental health problems among nonfatally injured people addressing emergency departments is higher than in the general population [1,2]. Some authors have studied the possible (causal) relationship between mental health and nonfatal injuries [3], but they did not consider specific psychopathological symptoms as issues associated with injuries. Nor did they study specific forms of nonfatal injuries, such as motor vehicle collisions (MVCs), for instance, evaluating the mental health of victims of (nonfatal/nonsuicidal) injuries in the particular developmental stage of adolescence. Yet, a bulk of studies have demonstrated that adolescence and puberty must be studied as peculiar developmental stages, which can be defined as challenging phases during which physical growth is quicker than in any other moment of life apart from prenatal months and early infancy [4,5]. Brain and cognitive functions also very rapidly mature, and relationships with peers gain a central role [6], fostering a reorganization of social and individual functioning [7,8]. These rapid neurobiological, social, and emotional modifications can be associated with a window of vulnerability to psychopathological risk, which can be expressed in behavioral, cognitive, and emotional dysregulation [9,10,11]. One of the most prevalent clinical manifestations in adolescence is characterized by binge eating behavior (BEb) [12] with frequent episodes of unusually large amounts of food intake without compensatory behaviors, which are associated with subjective experiences of feelings of loss of control and marked distress [13]. During adolescence, the youth experience rapid physical and neurobiological modifications that can be associated with increased concern and attention to body size, societal pressure for thinness, and preoccupation with peer acceptance. These factors can lead the youth to vulnerability to disordered eating, in general, and specifically to BEbs [14].

### 1.1. Binge Eating Behaviors (BEbs) and Motor Vehicle Collisions in Adolescence

Several authors in the Affect Regulation framework [15] have demonstrated that adolescence is a very liable developmental stage for the onset of mental health problems [16] and, as stated above, in particular, of disordered eating [17] that, with a prevalence of approximately 14%, is one of the most severe and common chronic conditions in this age group [18]. Previous research has shown that prevalence of eating disorders among adolescents is estimated to be 0.3% for anorexia nervosa, 0.9% for bulimia nervosa, and 1.6% for binge eating disorder, but subclinical problems are also frequent, with 2.5–4.6% of adolescents showing difficulties related to excessive food intake [19,20]. Accumulating research has also suggested that adolescents with eating disrupted behaviors show higher rates of access to emergency departments (EDpts) than the general population. In fact, Swanson and colleagues [21] have shown that 88% of adolescents with eating disorders have had at least one access to emergency departments, with 22% of adolescents having Binge Eating Disorder (BED). Specifically, sixteen percent of youth aged 14 to 20 years who accessed an EDpt had eating difficulties, even if not necessarily a full-blown condition [22]. Nevertheless, no specific research has been (to our best knowledge) expressly conducted to verify whether adolescent victims of road accidents are also likely to show disordered eating, one of the principal factors of vulnerability in this developmental age [23]. Yet, road collisions are a widely spread phenomenon, requiring even more attention when considering that, only in the United States, every year more than one million of 15- to 19-year-old subjects are involved in nonfatal road accidents [24] whereas in Italy MVCs constitute the first cause of death for people under 30 [25]. Many of these accidents do not result in subjects visiting emergency departments because no severe damage or traumas occur. Nevertheless, it has been shown that MVCs in adolescents’ lives are not isolated events occurring once in many years, but they can repeat several times in a relatively limited amount of time, usually with serious consequences [26]. Interestingly, it has been suggested that binge eating behaviors are frequently associated with peculiar clusters of psychopathological symptoms, which are also characteristic of young victims of traffic crashes [18,27]. Impulsivity, for instance, has been defined as a distinctive trait both of binge eaters and of MVC victims, as well as alexithymia and internalizing/externalizing problems [28]. However, no research has verified this overlap of maladaptive symptoms in adolescent victims of MVCs who showed binge eating behaviors. 

Why is it possible to hypothesize a specific link between MVCs and BEb? First, recent neurobiological studies focusing on adolescents with eating disorders demonstrated an altered functioning of the reward system, which is also connected with an impairment of risk perception and sensation seeking, which could result in risky driving behaviors and accidents [29]. Binge eating disorder has been demonstrated to be characterized by high impulsivity and emotion dysregulation [30]; thus, it is possible that adolescents with BEb show a high probability of MVCs. Second, some studies posited an impairment of the decision-making and attention processes in individuals with BEbs, which could be associated with inappropriate and dangerous behavioral choices while driving [31]. Third, other studies have shown connections between traffic crashes and BEb, focusing on the altered mental state experienced by subjects during compulsive eating episodes, with emotional and behavioral hyperactivation [32,33]. Fourth, it has been demonstrated that disordered eating (in particular comfort eating, which is typical of BEb clinical manifestation) is associated with risky driving (and therefore likely to be involved in road accidents) in adolescence [34]. It is evident that all the above conditions could constitute an even higher risk for adolescents who, even in normal conditions, may show emotional–behavioral dysregulation due to neurobiological immaturity and drive motorbikes (and not cars) due to age restrictions. 

### 1.2. The Present Study

In line with these considerations, and based on previous results [21], a research project was created in Italy in 2015 (as a part of the wider project started in 2000 named *Adolescents at Emergency Departments*) which aimed at studying BEbs and possible maladaptive psychological profiles in adolescent victims of MVCs, to deepen the understanding of the etiology of motor collisions and planning of effective preventive programs. Moreover, binge eating has been described as a silent problematic psychopathology in adolescence because not all BEb adolescents are overweight and they are therefore difficult to identify in the general population unless they personally ask for clinical help [35]. Indeed, weight gain can be a lengthy process, and adolescents may not be visibly overweight while already suffering psychological problems (as described in the previous paragraph), which could result in MVCs. For this reason, it appears sensible to administer a clinical screening for BEb in emergency departments to adolescent victims of traffic collisions, with the aim of its timely identification and effective treatment. In fact, early assessment and intervention with eating disorders is highly correlated with positive treatment outcomes, with improved odds especially for young patients [36,37]. 

Emergency centers, which regularly assist a large number of youths, are an ideal setting for developing health prevention programs as they do not necessitate youths to access a specialty mental health clinic so that adolescents at risk can be identified; this increases early detection and intervention, potentially reducing eating disorder onset [38]. 

In this project, only motorbike collisions are considered due to the age of participants (theoretical framework and empirical methodology are described elsewhere) [21]. Therefore, in the present paper all hypotheses, data, and conclusions refer to adolescent victims of motorbike collisions.

The aims of the present study were as follows: (1) identify specific clusters of maladaptive emotional-behavioral symptoms in adolescents involved in MVCs considering, alexithymia, impulsivity, and internalizing and externalizing problems; and (2) examine the prevalence of clinical binge eating symptoms and motor vehicle accidents in adolescents clustered on the basis of Objective 1. We hypothesize that higher levels of alexithymia, impulsivity, and internalizing and externalizing problems are associated with binge eating behaviors exceeding clinical cut-offs and higher frequency of past MVCs, identifying a group of multi-risk adolescents, for whom motorbike collisions represent just one aspect of a broader constellation of psychopathological risk. 

## 2. Method and Measures

### 2.1. Study Design and Setting 

A total of 226 adolescents aged 14 to 17 years in central Italy going to EDpts for medical care consequent to motorbike collisions from September 2014 to September 2015 were eligible for the study. All recruited subjects were drivers, not passengers, of the motorbike involved in the collision. Adolescents accessing EDpts for other traumatic events, suicidal attempts, sexual or physical abuse, altered mental status, or neurological impairment precluding informant consent were excluded from participation (*N* = 7). Patients who suffered severe injuries and cannot participate, non-Italian speaking subjects, and adolescents with no parent/guardian available for consent were also excluded (*N* = 13). Potential study participants were identified through patients’ tracking logs and recruited by researchers in the patients’ first treatment areas. The logs also reported alcohol and drug concentrations measured at the moment of the medical visit. Adolescents who tested positive for alcohol or drug use were excluded from the present study (*N* = 8). Furthermore, *N* = 39 adolescents were discharged before the researchers could approach them. Those who gave written consent were instructed to fill a 15-minute self-report questionnaire for screening the risk of binge eating disorder, which they independently completed (see below in the Measures section). The questionnaire also requested demographic information, including gender, age, and familial socio-economical status (SES). Following Bornstein et al.’s criteria [39], all participants showed an average SES. One hundred and fifty-nine adolescents (*N* = 159) were thus included in the present study.

In line with the Declaration Helsinki, the study was approved before its start by the Ethical Committee of the Psychology Faculty at Sapienza, University of Rome, with code: 4/2015_2, and all adolescents’ parents included in the study signed an informed consent form. Details that might disclose the identity of the subjects under study have been omitted. 

### 2.2. Measures

#### 2.2.1. Emotional and Behavioral Assessment

The Youth Self-Report (YSR) [40] (Italian version [41]) is a self-report questionnaire that covers a wide range of behavioral and emotional problems and also allows for assessing social skills. It contains 112 problem items, each to be rated on a 3-point scale (0 = not true, 1 = somewhat or sometimes true, 2 = very or often true), based on the past six months. Factor analyses yielded 8 syndromes, loading on two broad-band second-order factors: “Withdrawn”, “Somatic complaints”, and “Anxious/Depressed” syndromes comprise the “Internalizing” dimension, whereas “Rule-breaking” and “Aggressive” syndromes constitute the “Externalizing” dimension. Internalizing and externalizing scales are scored by summing the 0–1–2 ratings on items that are part of the included syndromes. The other three scales ("Social problems", "Thought problems", "Attention problems") and other items that were included in the “Other problems" subscale are considered for the computation of the Total Problem score. The YSR is an empirically validated and reliable measure with good internal consistency [40].

#### 2.2.2. Alexithymia 

The *20-item Toronto Alexithymia Scale* (TAS-20) [42] (Italian version [43]) is a self-report questionnaire made of 20 items that must be rated on a 5-point Likert scale, from 1 (strongly disagree) to 5 (strongly agree). The scale shows a three-interrelated-factor solution (difficulty identifying feelings, difficulty communicating feelings, and concrete thinking). A total score is computed and can be compared with cut-off scores, categorizing respondents into alexithymic (≥60), borderline (≤51 and ≥60), and non-alexithymic. The scale has demonstrated good reliability and validity in different cultures and clinical groups [42,44]. An Italian version for adolescents has been proposed [42], partially replicating the factor structure of the original TAS-20 and reporting higher levels of alexithymic traits in adolescents compared with in adults. 

#### 2.2.3. Impulsivity

The *Barratt Impulsiveness Scale* (BIS-11; [45]) is a self-report questionnaire designed to assess impulsivity and its components. Its 30 items are measured on a 4-point ordinal scale (from Rarely/Never to Almost Always/Always). A review of studies using BIS-11 on adult samples reported good psychometric proprieties [46]. Fossati and colleagues [47] proposed an adaptation of the scale for Italian adolescents and verified its psychometric properties. The scale showed good internal consistency (0.79), two-month test–retest reliability (0.89), and criterion-related validity, and demonstrated satisfying clinical usefulness.

#### 2.2.4. Binge Eating Behaviors

The Binge Eating Scale (BES) [48] (Italian version [49]) is a well-validated and widely used 16-item self-report questionnaire assessing the presence of BEbs, which may be indicative of an eating disorder. It has diffusely been used for the screening of binge eating symptoms in subjects accessing health and mental health services and in the general population [50]. It does not address a time frame and it is composed of a series of items, from which respondents select the statement that best describes their attitudes and behaviors. Scores of ≤17 were the cut-off for clinically relevant values. The BES has good test–retest reliability (r ¼ 0.87, *p* < 0.001) and it proved to discriminate binge eaters from non-binge eaters with high sensitivity (0.94) and high specificity (0.76) [51,52].

#### 2.2.5. Motor Vehicle Collisions 

Based on the number of adolescents’ visits to emergency departments because of motorbike collisions (data derived from patients’ logs), three categories were computed following Marcelli’s studies [53] on MVC recidivism in adolescence: (1) four or more past accidents; (2) three past accidents; and (3) 1 or 2 past accidents. 

#### 2.2.6. Statistical Analyses

Preliminarily, in order to have a normal standard distribution, we transformed the scores of the dimensions of emotional–behavioral functioning into Z-scores; specifically, the “Internalizing” and “Externalizing” dimensions of the YSR, the total score of alexithymia (TAS-20), and the total score of impulsivity (BIS-11) were considered. 

To identify clusters of maladaptive emotional–behavioral symptoms in MVC adolescents, we carried out hierarchical cluster analysis and multivariate analysis of variance (MANOVA) to test the explanatory power of the solution, followed by the examination of univariate main effects. A chi-square test was run to examine the prevalence of binge eating disorders and MVCs in the three clusters; eventually, the log-linear model was applied to analyze their interaction. Results were controlled for referred psychiatric comorbidities and prescribed medications. All analyses were performed with SPSS software (Version 21.0, IBM, Armonk, NY, USA).

## 3. Results

The participant average age was 15.5 years (SD = 0.91), ranging from 14 to 17 years. The sample consisted of 57% (*N* = 90) boys. Table S1 in the supplementary material shows binge eater and non-binge eater adolescents’ scores on the YSR, TAS-20 and BIS-11.

### 3.1. Clusters of Maladaptive Emotional–Behavioral Functioning in MVC Adolescents

A hierarchical cluster analysis was conducted using Ward’s method on squared Euclidian distances. We examined the pertinence of a three-cluster solution according to the following criteria: meaningfulness from a theoretical point of view, explanatory power (with the cluster solution explaining more than 50% of the variance), and parsimony. 

Multivariate analyses of variance (MANOVA) on the Z-scores of emotional–behavioral dimensions were performed in order to test the explanatory power of the solution. There was a significant multivariate main effect for MANOVA, Wilks’ λ = 0.051, *F* (8, 306.000) = 131.522, *p* < 0.001, partial eta squared = 0.775. Given the significance of the overall test, the univariate main effects were examined on the four emotional–behavioral dimensions considered in the study. Significant univariate main effects were obtained for each variable: Internalizing, *F* (2, 156) = 146.473, *p* < 0.001, partial eta squared = 0.653; Externalizing, *F* (2, 156) = 162.482, *p* < 0.001, partial eta squared = 0.676; total Alexithymia, *F* (2, 156) = 419.668, *p* < 0.001, partial eta squared = 0.843; total Impulsivity, *F* (2, 156) = 774.668, *p* < 0.001, partial eta squared = 0.909. For multiple comparisons, Bonferroni’s Post-hoc test was used (differences were considered statistically significant if *p* = 0.05/14 = 0.0036), indicating that all clusters differed significantly from each other in the emotional–behavioral dimensions. 

**Figure 1:**
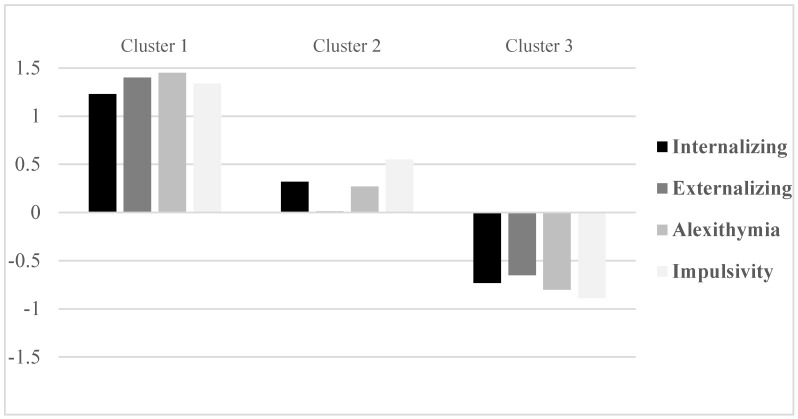
Z-scores for Internalizing, Externalizing, Alexithymia, and Impulsivity for the three clusters.

Adolescents in the first cluster (*N* = 37; 23.3%) had high scores on all four dimensions; a second cluster (*N* = 41; 25.8%) was composed of individuals with moderately high scores on Internalizing, Externalizing, Alexithymia, and Impulsivity. Adolescents of the third cluster (*N* = 81; 50.9%) scored low on all four measures. Figure 1 shows the Z-scores for Internalizing, Externalizing, Alexithymia, and Impulsivity for the three clusters, while Table 1 displays means and standard deviation values for the emotional–behavioral variables as functions of three clusters. Moreover, Table 1 reports the cut-off scores for YSR’s Externalizing and Internalizing dimensions, Alexithymia as measured by TAS-20, and Impulsivity as assessed by BIS-11, which may serve as clinically useful indexes. Cut-off scores were computed by summing the total sample’s means and 1 SD and ½ for each dimension; rounding off was applied. Table 1 also illustrates the percentages of subjects above the cut-off scores for each cluster; higher rates of individuals in Cluster 1 show scores above the cut-off (with rates ranging from 20 to 38%) than adolescents in the other two clusters (rates from 0 to 5%). 

### 3.2. Prevalence of Clinical BEbs and MVCs in the Three Clusters 

In order to verify how the three clusters relate to the presence/absence of the clinical symptoms of binge eating (measured through BES) and to the frequency of motorbike accidents, the chi-square test was performed. Results indicate that 100% of adolescents of Clusters 1 and 2 exceeded clinical cut-offs, while none of Cluster 3 did (X^2^ = 159.000, *p* < 0.001) (see Table 2). 

Concerning the number of accidents, categorized in three groups (4 or more, 3, and from 0 to 2 past accidents), 97.3% of adolescents in Cluster 1 reported 4 or more past accidents, while individuals of Cluster 2 reported 3 (53.7%) or less (41.5%) past collisions. Subjects of the third cluster had from 1 to 2 accidents in 55.6% of cases and 3 accidents in 32.1% of cases, showing a less clear distribution of frequency (X^2^ = 109.104, *p* < 0.001) (see Table 3). 

Given the significant association between the three-cluster solution and the number of accidents, the log-linear model was applied to analyze the parameters of their interaction. Results indicate that adolescents of Cluster 1 show a higher likelihood to be involved in MVCs (z = 3.392; *p* < 0.0001). Moreover, individuals of Clusters 1 and 2, with high and moderate levels of emotional–behavioral problems, respectively, show a higher probability to experience motorbike accidents than adolescents of Cluster 3, characterized by low levels of problematic traits ((z = −3.932; *p* < 0.0001) and (z = −3.958; *p* < 0.0001)). 

## 4. Discussion

The first aim of the present paper was to identify specific clusters of maladaptive emotional–behavioral symptoms (alexithymia, impulsivity, and internalizing and externalizing problems) in adolescent victims of MVCs [54]. Our results identified three different groups of adolescents: one group including adolescents with a high rate of internalizing and externalizing problems, alexithymia, and impulsivity; a second group of adolescents with moderate levels of emotional and behavioral problems in the above four dimensions; and a third group of youth with low scores in the four dimensions. In accordance with Cicchetti and Cohen [55], cluster analysis of psychopathological risk in a general population gave interesting results, identifying specific subgroups. This can constitute a very useful clinical tool for the planning of tailored secondary prevention and intervention programs to be applied to subjects at psychopathological risk who access emergency departments [56]. In this case, previous results were confirmed, with a three-partied cluster structure identifying groups with low, moderate, and high psychopathological risk [57]. Nevertheless, we were further interested in evaluating (in the three clusters) the prevalence of adolescents exceeding clinical cut-offs for the Italian population on Internalizing/Externalizing problems, Alexithymia, and Impulsivity. In fact, with the aim of identifying possible risk factors associated with multiple MVCs in adolescence, we needed to detect whether the cluster with more maladaptive scores was indeed characterized by clinical levels of psychological difficulties. Our results showed that only the first cluster of adolescents exceeded cut-offs with a significant rate (ranging from 20 to 38%). Thus, not only did the other two clusters of grouped adolescents have lower scores on the four maladaptive dimensions, but they also sorted subjects who were not at risk of psychological malfunctioning. 

Based on [22] who linked adolescents’ disordered eating to their emergency department access, our second aim was to evaluate the prevalence of clinical BEbs and MVCs in the three clusters. Results showed that all adolescents of Clusters 1 and 2 had clinical levels of BEbs, while none of Cluster 3 did. Moreover, with regards to the number of MVCs, results showed that adolescents in Cluster 1 had a higher likelihood to be involved in MVCs than adolescents of Clusters 2 and 3, which were characterized by lower maladaptive scores.

In sum, our results identified three different groups of adolescents: a first group including adolescents with clinical levels of BEb and characterized by a high rate of past motor vehicle accidents and high levels of internalizing and externalizing problems, alexithymia, and impulsivity; a second group of adolescents with clinical levels of BEb, characterized by an intermediate number of collisions and intermediate levels of emotional and behavioral problems in the above four dimensions; and a third group of youth without clinical levels of BEb, with an intermediate number of experienced accidents and with low scores in the four dimensions. This latter group differentiates itself from the second one by the lower levels of internalizing, externalizing, alexithymia, and impulsive problems, rather than by the number of motorbike collisions. These results are consistent with previous literature in that it is confirmed that clinical binge eating symptoms are associated with a broader configuration of maladaptive psychological difficulties, specifically with those symptoms impairing the capacity to regulate impulses and to identify and communicate feelings [56,57]. However, our study is the first to demonstrate this in MVC adolescents. A bulk of studies has hinted that BEbs might be considered as an active (maladaptive) strategy to escape from self-awareness, acted upon when the subject is in a condition of emotional distress or to numb the sense of repugnance that the binge often causes in the patient [58,59]. Contemporarily, it has also been shown that patients with eating difficulties who access healthcare resources complain of physical symptoms [60] that they cannot directly recognize as connected with their maladaptive symptoms in the area of eating [61,62]. While the results of this study do not allow us to draw conclusions, we speculate that the awareness of the nature and etiopathogenesis of symptoms in patients with eating disrupted behaviors could be weak (notwithstanding the severe psychological sufferance), and that access to EDpts could constitute an attempt to have the organic and psychological areas connected by the intervention of health professionals. Different from adolescents who show clinical BEbs, who fail in regulating their emotions and surrender to the impulse of binging, youth involved in traffic accidents could not be cognitively aware of their maladaptive response to psychological distress and in this sense could activate a more "passive" strategy to cope with it, becoming victims of road accidents [57]. However, in this perspective, both BEb adolescents and youths in MVCs could use the emergency department visits as unconscious attempts to receive psychological help. 

Our study has some limitations. First, although we carried out our study by gathering longitudinal data, at the moment we cannot establish causal links between adolescents’ psychopathological risks, clinical binge eating symptoms, and the number of motor vehicle accidents. Second, we did not consider in this paper information coming from adolescents’ parents (e.g., family functioning, parental psychopathological risk, etc.), which may contribute to the understanding of family dynamics [63]. For instance, Bingham and Shope [64] found that adolescent victims of traffic accidents were more likely than their peers to have permissive parents and a low level of parental control. Moreover, Taubman-Ben-Ari and Katz-Ben-Ami [65] showed that a negative family climate is correlated with adolescents’ proneness to road accidents. This information was unattainable in the context of the emergency department because the arrangements with hospitals did not include administering a large protocol of instruments, so we chose to concentrate on adolescents’ individual characteristics since we assessed other aspects elsewhere. Third, the sample homogeneity in terms of race and geographical origin does not enable wide generalizations of the results to the general population to be made.

Notwithstanding these limitations, our study has several strengths. This is the first study, to our best knowledge, to evaluate a possible association between MVCs and binge eating symptoms in adolescents visiting emergency departments, conjugating clinical experience and empirical research. Emergency departments hold a significant role in early recognition of adolescents with disordered eating. In fact, after the assessment, they can advise and refer youths to appropriate medical and psychological services, potentially reducing maladaptive eating behavior recidivism, and produce improved outcomes among these patients. These findings highlight the importance of screening for BEbs among adolescents with frequent access to EDpts unrelatedly to their stated reason for the visit [18]. Moreover, all measures used were widely validated, even in such stressful settings as EDpts [66,67,68,69,70].

## 5. Conclusions

The results of this study suggest that adolescents’ motor collisions could be associated with their difficulties in emotion regulation and with their impaired psychological profiles, which could also underpin their disordered eating. The identification of specific clusters of psychopathological symptoms among this population could be useful for the construction of prevention and intervention programs aimed at reducing motor collision recidivism and alleviating co-occurring psychopathologies.

## Figures and Tables

**Table 1:** Means (SD) using the three-cluster solution, cut-off scores, and percentages of individuals above cut-off.

Dimensions	Cluster 1		Cluster 2		Cluster 3		Cut-Off Score (Rounded)	Cluster 1	Cluster 2	Cluster 3
	M	SD	M	SD	M	SD				
Internalizing	37.00	4.89	27.15	8.31	15.73	5.98	39.93 (40)	20%	5%	0%
Externalizing	26.22	3.04	16.32	6.20	11.67	2.99	26.90 (27)	38%	5%	0%
Alexithymia	65.19	4.85	46.73	8.34	30.02	5.47	65.91 (66)	35%	0%	0%
Impulsivity	98.70	8.19	81.51	6.69	50.19	5.75	98.13 (98)	21.6%	0%	0%

**Table 2:** Percentages of individuals with clinically relevant binge eating behaviors as for each cluster.

Cluster	NO % (*N*)	YES % (*N*)	Total % (*N*)
1	0 (0)	100 (37)	100 (37)
2	0 (0)	100 (41)	100 (41)
3	100 (81)	0 (0)	0 (0)
Total % (N)	50.94 (81)	49.06 (78)	100 (159)

**Table 3:** Distribution of individuals according to accident number as a function of cluster.

Cluster	Number of Past Accidents		
	≥4% (*N*)	=3% (*N*)	1 or 2% (*N*)
1	97.30 (36)	2.70 (1)	0 (0)
2	4.88 (2)	53.66 (22)	41.46 (17)
3	12.35 (10)	32.10 (26)	55.56 (45)
Total % (*N*)	30.19 (48)	30.82 (49)	38.99 (62)

## Supplementary Materials

The following are available online at http://www.mdpi.com/1660-4601/15/2/355/s1, Table S1: Comparison between binge-eating and non-binge-eating adolescents.
