# Supplementary material for: A Theoretical and Empirical Linkage between Road Accidents and Binge Eating Behaviors in Adolescence
Source: Int J Environ Res Public Health. 2018 Feb 17;15(2):355. doi: 10.3390/ijerph15020355 (PMC5858424; doi:10.3390/ijerph15020355)
Supplement: Supplementary file 1 [file ijerph-15-00355-s001.docx]

**Supplementary Materials**

**Table S1.** Comparison between binge-eaters and non-binge-eaters adolescents.

| **Dimensions** | **Binge-eaters**  **(*N* = 78)** | | **Non binge-eaters**  **(*N* = 81)** | |  | |
| --- | --- | --- | --- | --- | --- | --- |
|  | M | SD | M | SD | *t or χ2* | *p* |
| Age | 15.38 | 0.82 | 15.56 | 0.91 | -1.24 | .203 |
| Sex: male | 51.3% |  | 61.7% |  | 0.18 | .217 |
| Emotional-behavioral total score | 82.19 | 17.6 | 48.25 | 11.28 | 14.48 | < 0.001 |
| Internalizing symptoms | 33.10 | 10.2 | 15.73 | 5.98 | 13.78 | < 0.001 |
| Externalizing symptoms | 21.01 | 7.00 | 11.67 | 2.99 | 11.02 | < 0.001 |
| Alexithymia total score | 55.49 | 11.53 | 30.02 | 5.47 | 17.88 | < 0.001 |
| Difficulty identifying feelings | 22.22 | 6.21 | 9.54 | 1.81 | 17.60 | < 0.001 |
| Difficulty communicating feelings | 9.79 | 2.99 | 10.26 | 2.31 | −1.09 | .275 |
| Concrete thinking | 23.47 | 7.56 | 10.22 | 4.42 | 13.54 | < 0.001 |
| Impulsivity total score | 77.31 | 4.93 | 65.23 | 4.51 | 16.11 | < 0.001 |
| Attentional impulsivity | 21.62 | 2.36 | 15.10 | 2.45 | 17.05 | < 0.001 |
| Motor impulsivity | 31.03 | 3.92 | 19.74 | 2.67 | 21.26 | < 0.001 |
| Non-planning impulsivity | 24.67 | 3.55 | 30.40 | 3.47 | -10.26 | < 0.001 |

The two groups do not show significant differences on demographic characteristics of age and sex. The profile of binge-eater significantly differs from the non-binge-eater group in terms of emotional-behavioral problems, alexithymia and impulsivity. Results remain significant when the Bonferroni correction (*p* = .05/14 = 0.036) is used.
